# Supplementary material for: Gallium nitride catalyzed the direct hydrogenation of carbon dioxide to dimethyl ether as primary product
Source: Nat Commun. 2021 Apr 16;12:2305. doi: 10.1038/s41467-021-22568-4 (PMC8052344; doi:10.1038/s41467-021-22568-4)
Supplement: Supplementary file 1 — Supplementary Information [file 41467_2021_22568_MOESM1_ESM.pdf]

Supplementary Information

Supplementary discussion

Detailed structural characterizations. The XPS spectra of fresh and spent GaN catalysts are shown in Supplementary Fig. 1, and the corresponding binding energies (B.E.) are listed in Supplementary Table 1. Over the fresh GaN-10.5, GaN-16.7 and GaN-26.6 catalysts, B.E. for the Ga 2*p* spectra (Supplementary Fig. 1a) are corresponding to Ga $2p_{3/2}$ and Ga $2p_{1/2}$ of the wurtzite-structure GaN [1]. This is consistent with the results of Ga 3*d* spectra (Supplementary Fig. 1c), in which the peaks centered at 19.8 and 20.0 eV are attributed to the Ga-N bonds [1,2]. However, over the fresh GaN-7.4 catalyst, the Ga 2*p* spectrum (Supplementary Fig. 1a) shifts to a lower B.E., indicating the presence of Ga oxide [3]. A more direct evidence is proposed in the Ga 3*d* spectrum (Supplementary Fig. 1c), in which the peak corresponding to Ga_2O_3 is detected at 21.1 eV [4]. This is possibly because that the nitration is incomplete for GaN-7.4 due to the shorter duration of the calcination. Furthermore, the N 1*s* spectra (Supplementary Fig. 1e) confirmed the GaN phase on the surface of catalysts, i.e. the B.E. at 397.4–397.6 eV attributed to N in the Ga-N bond [2]. The shoulder appeared at around 395.6 eV is from the Ga LMM Auger peak [2]. Noteworthy, only slight shifts within 0.2 eV are observed for the XPS spectra of the spent catalysts (Supplementary Figs. 1b, d and f), and no additional peak is detected, indicating that the GaN phase on the surface of catalysts are stable during the CO_2 hydrogenation.

The $\chi(\kappa)$ spectra and the corresponding Fourier transform magnitudes of the $\chi(\kappa)$ spectra over GaN-7.4 were fitted using the model for the wurtzite-structure GaN (a = 3.18 Å, c = 5.168 Å) constructed from the references [5,6]. In the Fourier transformed spectra (Supplementary Fig. 3b), the three nearest neighboring shells consist of 4 N atoms at 1.92 Å, 12 Ga atoms at 3.18 Å and 12 N atoms at 3.69 Å, which are close to those in the spectra of the standard wurtzite-structure GaN. Thus, GaN-7.4 is the typical wurtzite structure, which is consistent with the results of XRD and TEM.

The TEM images for different GaN catalysts are shown in Supplementary Fig. 4.

Irrespective of the samples, the exposed crystalline planes are exclusively (001), (100), and (110). The (001) plane shows the fringe spacing of 0.28 nm [7]. However, in the case of the (110) and (100) crystalline planes, the same lattice spacing of 0.26 nm is observed [7,8]. Moreover, the fringe spacing for each exposed plane is independent on the crystal size of the GaN catalyst, indicating the high crystallinity of the GaN catalysts. Therefore, the TEM results reveal the well-crystallized wurtzite-structure GaN.

Acidity of GaN catalysts. As indicated from the results in Supplementary Fig. 6a and Supplementary Table 3, the total amount of acid sites over the fresh GaN-26.6 (27.3 $mmol_{NH3}$/g) is almost doubled that over the spent catalyst (13.6 $mmol_{NH3}$/g). This indicates the gradual loss of the acidity with increasing the time on stream, and is consistent with the decreased CO_2 conversion during the initial induction period. Moreover, the secondary reactions of DME and/or methanol to HCs can be inhibited due to the loss of the acidity, leading to the increased DME selectivity and the decreased selectivity of HCs during the induction period (Figure 4).

As shown in Supplementary Fig. 9, all of the GaN catalysts show a band at ~1456 cm^{-1}, which is assigned to the pyridine adsorbed on Lewis acid sites. Moreover, the band at ~1541 cm^{-1} is assigned to the pyridine adsorbed on Brønsted acid sites. Noteworthy, the GaN-26.6 catalyst shows a very weak IR band at ~1541 cm^{-1}, indicating its very low Brønsted acidity. To quantify the amounts of Lewis and Brønsted acid sites, the bands at ~1456 and ~1541 cm^{-1} are integrated, respectively, by following the method reported by Emeis [9]. As shown in Supplementary Fig. 5, the density of Lewis acids is decreased in the order of GaN-7.4 > GaN-16.7 > GaN-26.6 > GaN-10.5 while the density of the Brønsted acids is decreased in the order of GaN-7.4 > GaN-10.5 > GaN-16.7 >> GaN-26.6. As a result, the total amount of acid sites is decreased in the order of GaN-7.4 > GaN-16.7 > GaN-10.5 > GaN-26.6.

Comparison of catalytic performance with hybrid catalysts. Although the hybrid catalysts exhibit a higher space-time yield of DME (STY_{DME}) at lower reaction temperatures of 240–260 ºC than the GaN catalyst under a higher temperature of 360 ºC, both the CO-free selectivity of DME and STY_{DME} over Cu-based hybrid catalysts are significantly decreased with increasing the reaction temperature (Supplementary Table 4). As a result

of the much higher CO-free selectivity of DME over the GaN catalyst than that over the hybrid catalysts under similar reaction conditions, a slightly higher STY_{DME} is obtained over the GaN catalyst, and the $CaCO_3$-GaN catalyst shows considerably higher STY_{DME} than the traditional hybrid catalysts. Thus, the CO_2-to-DME reaction at a higher temperature is more favorable over the GaN catalyst than that over the Cu based hybrid catalysts.

Results of DME or MeOH as a reactant over GaN-26.6. In a quartz fixed-bed reactor (id = 10.0 mm), the experiments by using DME + H_2O, only MeOH, or MeOH + H_2O as reactants were performed, respectively. The flow rates of DME and the N_2 diluent were controlled by a mass flow controller, and methanol and H_2O were pumped into the reactor by a P230 pump of Elite Dalian Company. The reactions were conducted under the conditions of P = 0.1 MPa, T = 320 or 360 °C, and GHSV = 4000 $mL \cdot g^{-1} \cdot h^{-1}$. For co-feeding DME and H_2O, the molar ratio of DME/H_2O/N_2 was 1/4/5. The molar ratio of MeOH/N_2 of 1/9 was used for the experiments of the methanol dehydration. When MeOH and H_2O were co-fed as reactants, the molar ratio of MeOH/H_2O/N_2 was kept at 1/4/5. The reaction mixture was analyzed by the on-line GC-9560 gas chromatograph (Huaai Company) equipped with TCD and FID detectors. The effluents of N_2, H_2, CO, and CO_2 were separated by a 5A molecular sieve column and were analyzed by TCD. The separation of DME, MeOH, and C_{1-5} HCs were performed on a Plot-Q column (Bruker) and were detected by FID. The conversion of DME or MeOH and the selectivity of different products were calculated with the following equations:

$$\text{DME/MeOH conversion} = [F_{in}(\text{DME/MeOH}) - F_{out}(\text{DME/MeOH})] / F_{in}(\text{DME/MeOH}) \times 100\%$$

$$\text{Selectivity of } A = F_{out}(A) / [\text{F}_{in}(\text{DME/MeOH}) - F_{out}(\text{DME/MeOH})] \times 100\%$$

where F_{in} is the flow rate in the inlet and F_{out} is the flow rate in the outlet. A is one of the products detected by TCD or FID.

As shown in Supplementary Fig. 8a, methanol is the only product at a reaction temperature of 320 ºC, indicating the hydrolysis of DME ($CH_3OCH_3 + H_2O = 2CH_3OH$) occurs exclusively over the GaN catalyst. However, when the reaction is performed at a high temperature of 360 ºC, a comparable selectivity of methanol and CO_2 is obtained together with the production of H_2 (Not show), indicating that both the hydrolysis of DME

and the steam reforming of DME (SRD, $CH_3OCH_3 + 3H_2O = 2CO_2 + 6H_2$) occur in a comparable extent. When only methanol is fed as the reactant (Supplementary Fig. 8b), irrespective of the reaction temperatures, DME from the dehydration of methanol ($2CH_3OH = CH_3OCH_3 + H_2O$) is always the main product. Moreover, a higher reaction temperature increases the selectivity of both CO and CO_2. Thus, the steam reforming of methanol (SRM, $CH_3OH + H_2O = CO_2 + 3H_2$) and the decomposition of methanol ($CH_3OH = CO + 2H_2$) occurs significantly at a higher reaction temperature, which is confirmed from the simultaneous release of H_2 (Not show). On the contrary, when methanol and H_2O are co-fed with a molar H_2O/methanol ratio of 4 (Supplementary Fig. 8c), the selectivity of DME becomes very low, i.e. 0.20 and 0.16% at the reaction temperatures of 320 and 360 ºC, respectively. Moreover, CO and CO_2 are the main products irrespective of the reaction temperatures, and the selectivity of CO is significantly increased with increasing the reaction temperature. Considering that H_2O is the product for the dehydration of methanol and the reactant for the SRM reaction, this observation is well understandable, i.e., the inhibiting effect of the added H_2O on the dehydration of methanol vs. the increased SRM rate with the increased partial pressure of H_2O. In fact, SRD ($CH_3OCH_3 + 3H_2O = 2CO_2 + 6H_2$) is essentially the reverse reaction of the hydrogenation of CO_2 to DME, and a two-step mechanism, i.e., the hydrolysis of DME to methanol followed by the steam reforming of the intermediate methanol is generally accepted. In this case, the hybrid of the supported Cu and solid acid is a good catalyst for SRD. If it is interested, please see the detailed discussion on the SRD catalytic network in our works, e.g., Chemical Engineering Journal 187 (2012) 299-305 and Catalysis Today 351 (2020) 68-74.

Thus, as a result of the acidity (Supplementary Fig. 6), the GaN catalysts can catalyze both the hydrolysis of DME and the dehydration of methanol.

Discussion on the DRIFTS results. Supplementary Fig. 11 shows the operando DRIFTS for the hydrogenation of CO_2 over GaN-26.6 at 360 ºC and 0.1 MPa, and the assignment of the IR bands based on the related references is summarized in Supplementary Table 6. For directly reflecting the change for the intensity of the IR bands, the time-on-stream intensity of different IR bands is plotted (Supplementary Fig. 12).

At the beginning of the reaction, the band for carboxylate species (COO^*) is detected at 1263 cm^{-1} [9,10]. However, this band is vanished after a TOS of 5 minutes (Supplementary Fig. 12). The IR bands at 1303 and 3014 cm^{-1} assigned to the methyl ($^*CH_3$) [12,13] are strong at the beginning of the reaction. However, the intensity of the two peaks decreases significantly at a TOS of 5 min, and it is kept at a low level until the end of the test (Supplementary Figs. 11 and 12). Because of the interruption by the gaseous CO_2, the formation of hydroxyl species ($^*OH$) at 3600 cm^{-1} [14] cannot be discussed. In the case of the IR band at 1339 cm^{-1}, it can be assigned to monodentate carbonate species (CO_3^{2-}) according the reference [12]. These results indicate that the CO_2 molecules are activated over the GaN surface in the form of COO^*, and the subsequent dissociation of the C-O bonds assisted by hydrogen may lead to the formation of the adsorbed methyl group ($^*CH_3$). Simultaneously, the adsorbed oxygen atoms may be consumed by hydrogen to form the adsorbed OH or H_2O. Alternatively, it can also be reacted with the adsorbed CO_2 to give CO_3^{2-} species. However, the CO_3^{2-} species are hardly detected after a TOS of 5 minutes, indicating that CO_3^{2-} is not a stable intermediate on the GaN catalysts.

The IR band at 1430 cm^{-1} assigned to bicarbonate species (HCO_3^-) [15,16] is detected after a TOS of 3 minutes. It may be formed via two routes, i.e. 1) the hydrogenation of the CO_3^{2-} species [12,16,17,18], which is supported from the decreased intensity of the CO_3^{2-} species together with the increased intensity of the HCO_3^- species after a TOS of 3 minutes (Supplementary Fig. 12); 2) the insertion of CO_2 into the Ga-OH [19,20]. Considering the low stability of the CO_3^{2-} species over the surface of GaN, it is speculated that the HCO_3^- species may mainly be formed via route 1 at the beginning of the reaction and via route 2 at the later stage of the reaction.

The IR bands at 1606 and 1395 cm^{-1} can be ascribed to the asymmetric (ν_{as}) and symmetric (ν_s) stretching vibrations of the O-C-O bonds of $HCOO^*$ species, respectively [21,22]. Moreover, the difference between the wavenumbers of ν_{as} and ν_s ($\Delta\nu = \nu_{as} - \nu_s$) is 211 cm^{-1}. According to the reference reports [14,23,24], $\Delta\nu$ between 200 and 250 cm^{-1} is originated from the bidentate formate. Thus, over the GaN surface, $HCOO^*$ species may be adsorbed in a bidentate mode as shown in Supplementary Table 6. The formate may be produced from the hydrogenation of COO^* or CO_3^{2-} species since that the continuous

increase in the intensity of the IR absorbance of $HCOO^*$ with TOS is accompanied with the decreased IR absorbance of COO^* and CO_3^{2-}.

Finally, the IR band at 1456 cm^{-1} appears after about 5 minutes, and its intensity is slightly increased with increasing the TOS (Supplementary Fig. 11). Although the band is partially overlapped with the absorbance of HCO_3^-, it can be assigned to the characteristic C-H bond vibrations of the absorbed DME according to the reference [25].

In the case of the methoxyl (CH_3O^*) species, its characteristic IR band appears at 1050–1060 cm^{-1} in the operando DRIFTS as reported in the reference [26]. However, in our case, no clear peak at around 1050 cm^{-1} is observed (Supplementary Fig. 11), indicating that CH_3O^* species are very possibly not the intermediate for the formation of DME.

Detailed DFT results. The calculated lattice parameters of the hexagonal GaN crystal, i.e., a = 3.12 Å and c = 5.09 Å, are close to the experimental values[27]. The GaN(110) surface is represented by a five-layer slab with a 2 × 2 supercell, and a vacuum of 15 Å was used to separate the slabs as shown in Supplementary Fig. 13. The bottom two layers were fixed in their bulk position while the top three layers with the adsorbates were free. The GaN(100) surface is represented by a six-layer slab with a 3 × 2 supercell, and a vacuum of 15 Å was used to separate the slabs as shown in Supplementary Fig. 13. The bottom two layers were fixed in their bulk position while the top four layers with the adsorbates were free. The GaN(120) surface, the (110)/(100) interface over GaN, is represented by a six-layer slab with a 2 × 2 supercell, and a vacuum of 15 Å was used to separate the slabs as shown in Supplementary Fig. 13. The bottom two layers were fixed in their bulk position while the top four layers with the adsorbates were free. The N atom-terminated GaN(001) surface is represented by an eight-layer slab with a 3 × 3 supercell, and a vacuum of 15 Å was used to separate the slabs as shown in Supplementary Fig. 13. The bottom four layers were fixed in their bulk position while the top four layers with the adsorbates were free.

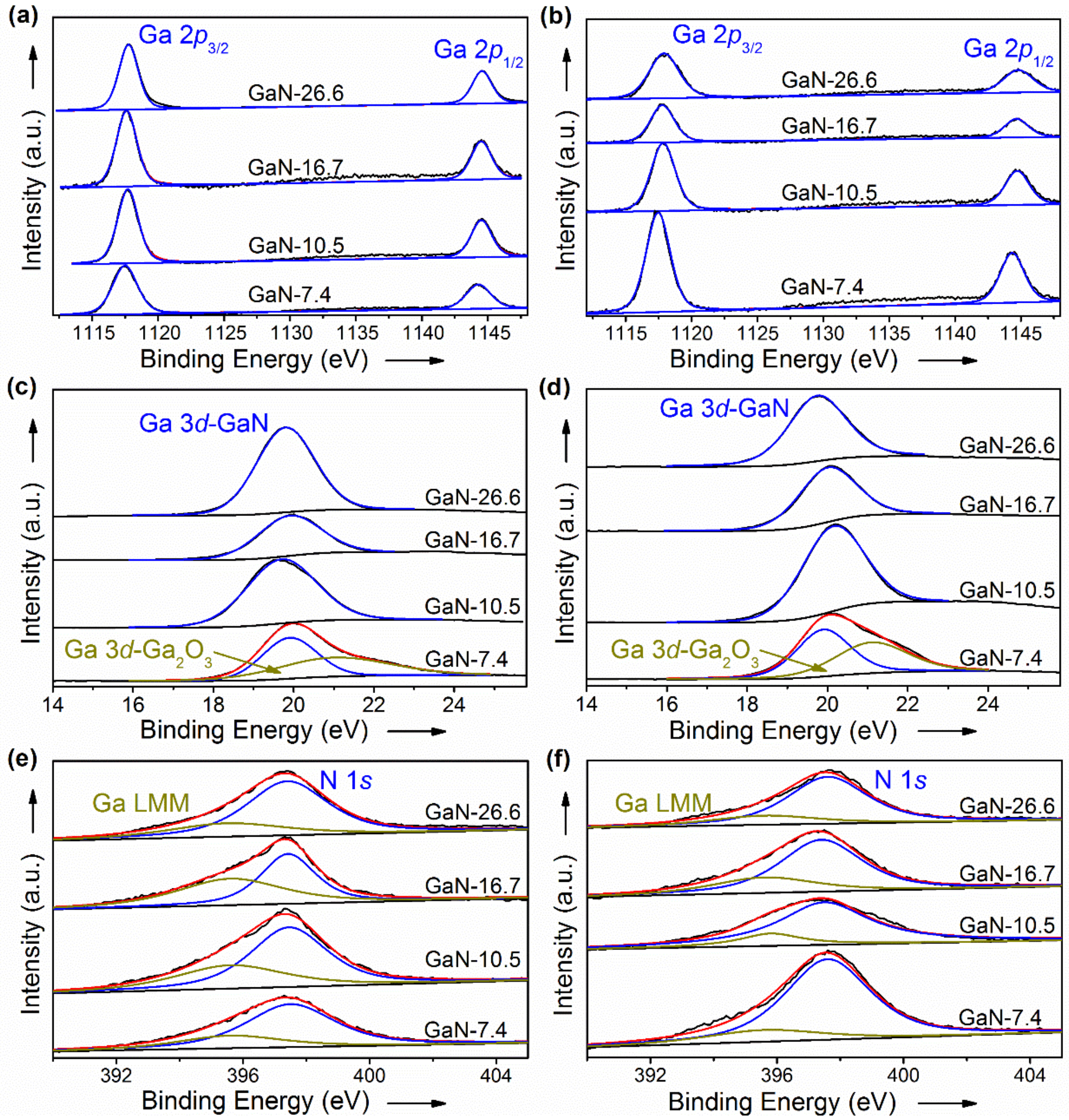

Supplementary Fig. 1. XPS spectra over the fresh and spent GaN catalysts. **(a)** Ga 2*p* spectra over the fresh catalysts. **(b)** Ga 2*p* spectra over the spent catalysts. **(c)** Ga 3*d* spectra over the fresh catalysts. **(d)** Ga 3*d* spectra over the spent catalysts. **(e)** N 1*s* spectra over the fresh catalysts. **(f)** N 1*s* spectra over the spent catalysts.

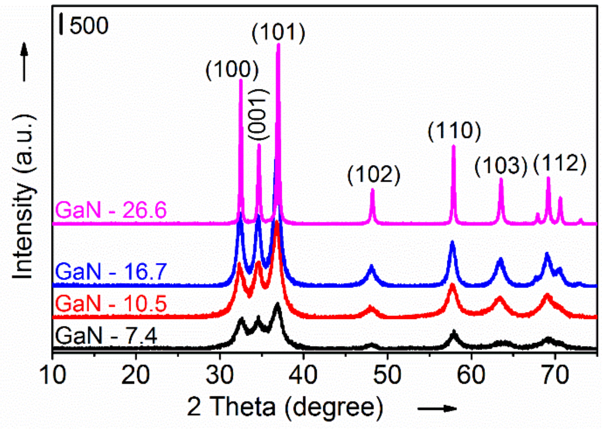

Supplementary Fig. 2. XRD patterns of different GaN samples. The different crystal planes are identified with JCPDS = 65-3140 as the reference.

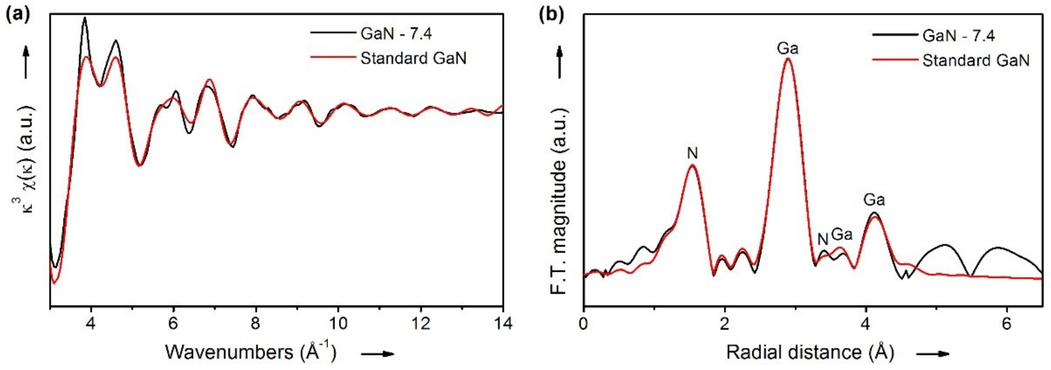

Supplementary Fig. 3. XAFS of Ga κ-edge on GaN-7.4 and a standard GaN. **(a)** The χ(κ) spectra; **(b)** Fourier transform (F.T.) magnitudes of the χ(κ) spectra.

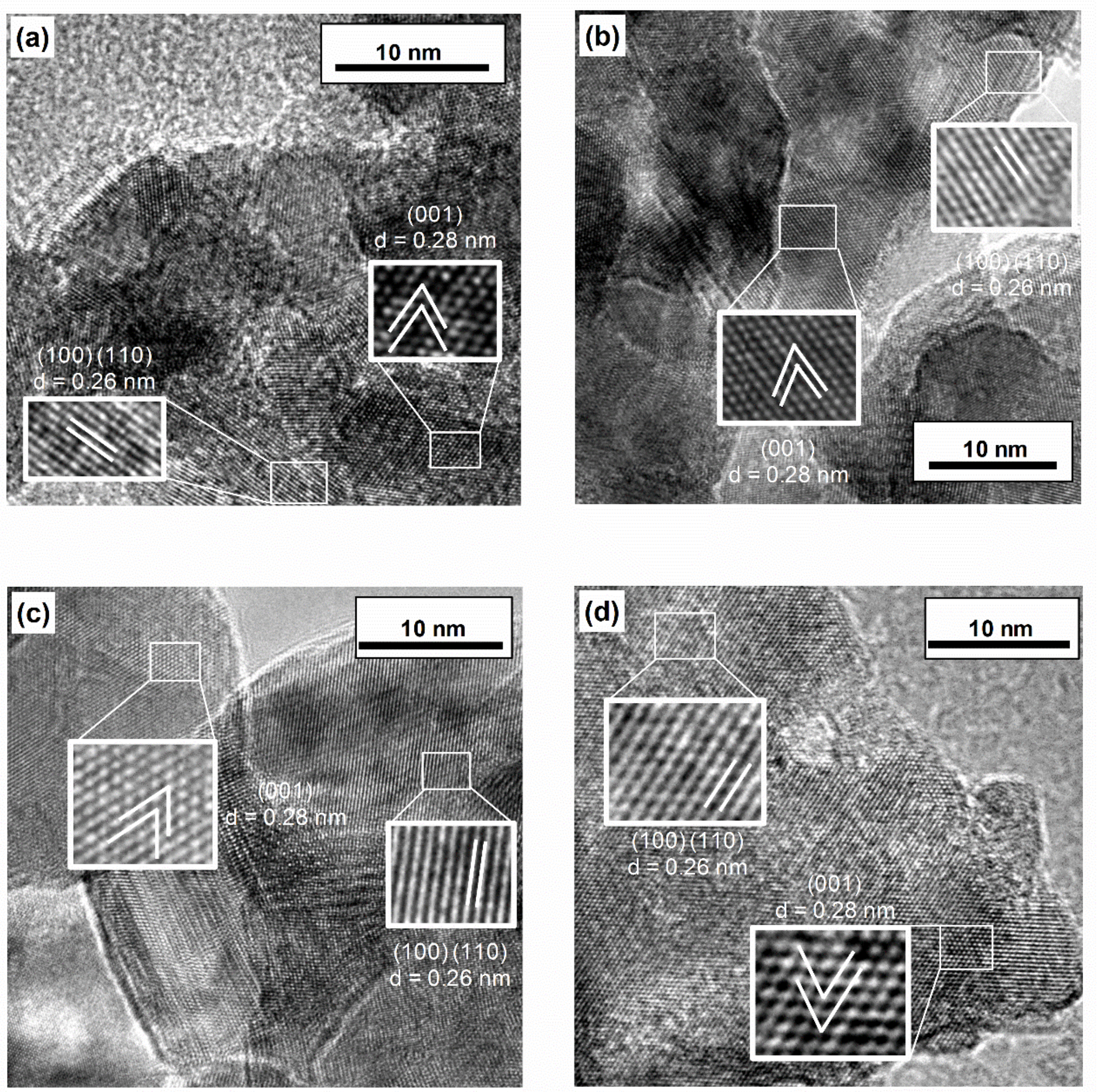

Supplementary Fig. 4. TEM images over the different GaN catalysts. **(a)** GaN-7.4. **(b)** GaN-10.5. **(c)** GaN-16.7. **(d)** GaN-26.6.

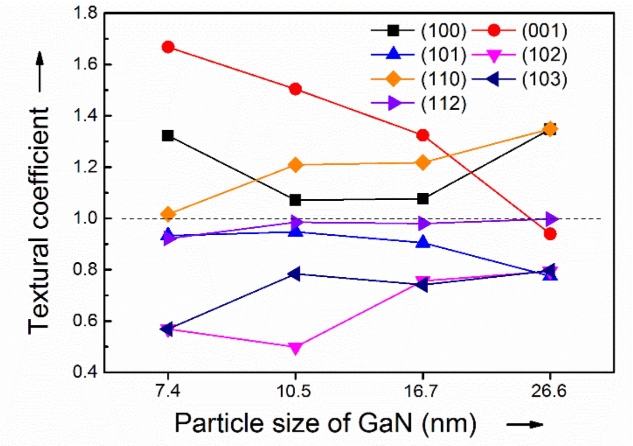

Supplementary Fig. 5. Textural coefficients for different planes of GaN with different crystal sizes. The textural coefficients and crystal sizes are determined based on the XRD patterns in Supplementary Fig. 2.

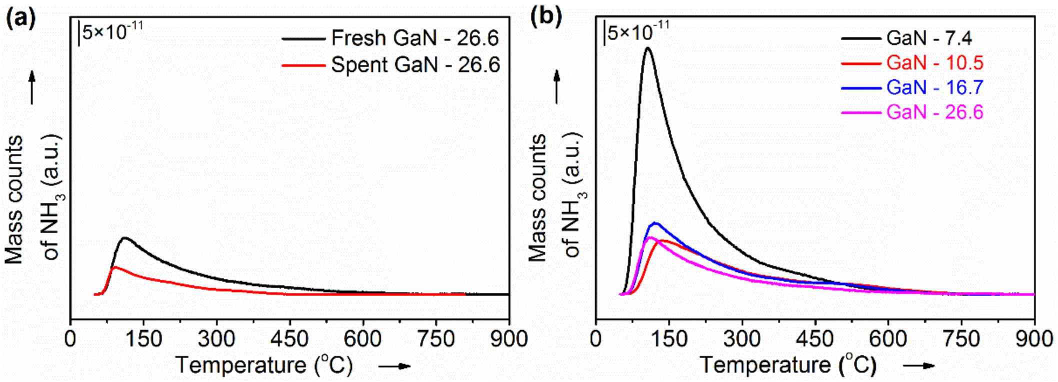

Supplementary Fig. 6. NH_3-TPD profiles. **(a)** Over the fresh and spent GaN-26.6 catalysts. **(b)** Over the fresh GaN catalysts with different crystallite sizes. The spent GaN-26.6 catalyst was obtained under the conditions of *P* = 2.0 MPa, *T* = 360 ºC, H_2/CO_2 = 2, gas hourly space velocity = 3000 mL·g^{-1}·h^{-1} and a time on stream of 100 h.

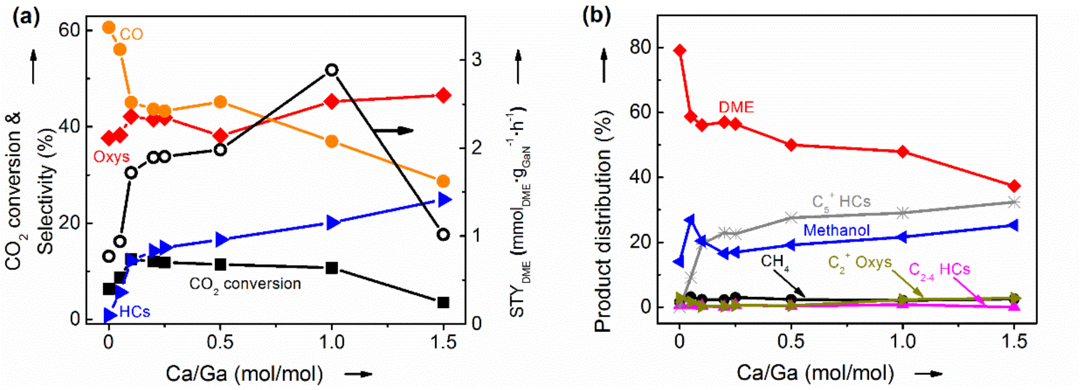

Supplementary Fig. 7. Effect of $CaCO_3$/GaN-26.6 molar ratio on the hydrogenation of CO_2. **(a)** The CO_2 conversion, selectivity of CO/HCs/Oxys, and the space–time yield (STY) of dimethyl ether (DME). **(b)** The distributions of hydrocarbons (HCs) and oxygenates (Oxys). Reaction conditions of T = 360 ºC, P = 2.0 MPa, gas hourly space velocity = 3000 mL·g^{-1}·h^{-1}, H_2/CO_2 = 2, and time on stream = 40 h.

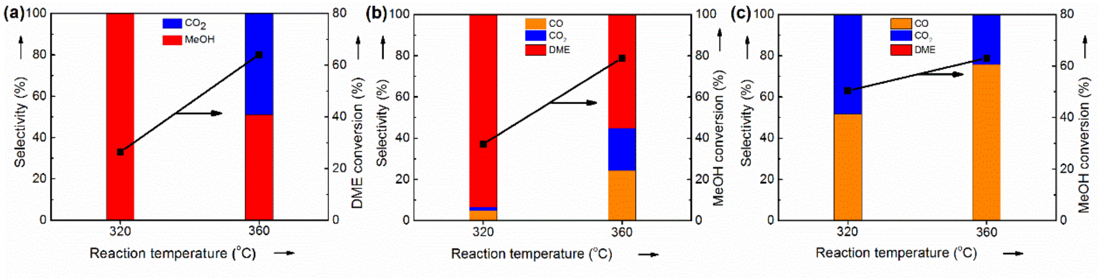

Supplementary Fig. 8. Results using DME or MeOH as a reactant over GaN-26.6. The conversion and the selectivity of different products are present for the experiments with the reactants of **(a)** DME + H_2O, **(b)** only MeOH, and **(c)** MeOH + H_2O under the conditions of T = 320 or 360 ºC, P = 0.1 MPa, and gas hourly space velocity = 4000 mL·g^{-1}·h^{-1}.

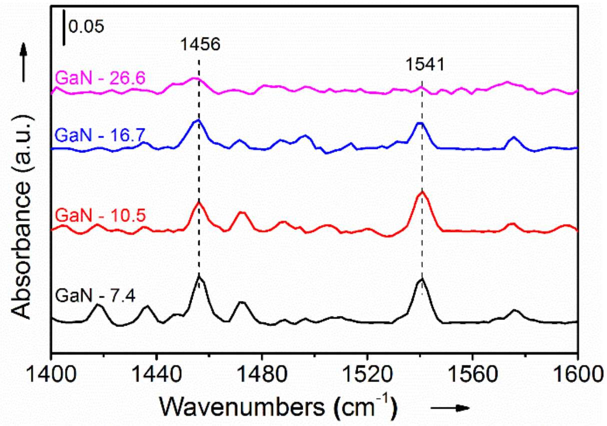

Supplementary Fig. 9. Pyridine-IR spectra of different GaN catalysts. The pyridine is absorbed at 30 °C and desorbed at 150 °C.

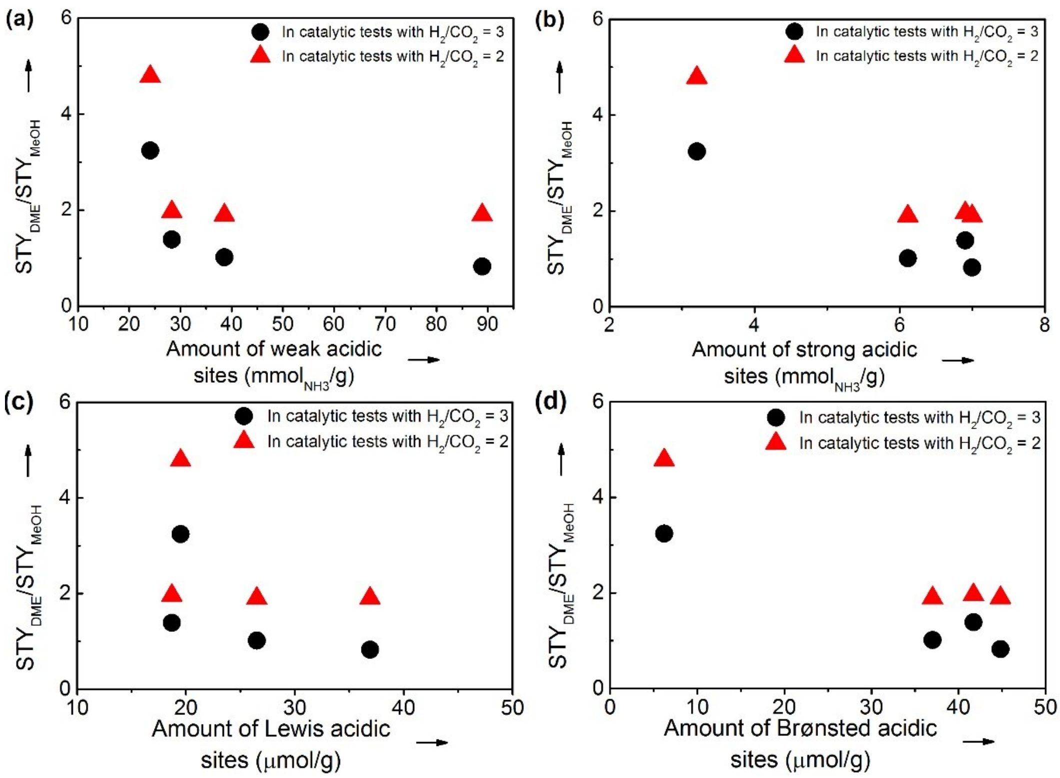

Supplementary Fig. 10. The correlation between STY_{DME}/STY_{MeOH} and the acidity of GaN. **(a)** As a function of the amount of weak acid sites. **(b)** As a function of the amount of strong acid sites. **(c)** the amount of Lewis acid sites. **(d)** As a function of the amount of Brønsted acid sites. The amounts of weak and strong acidic sites were measured according to the NH_3-TPD profiles. The STY was measured for the CO_2 hydrogenation under the conditions of T = 360 ºC, P = 2.0 MPa, gas hourly space velocity = 3000 mL·g^{-1}·h^{-1}, H_2/CO_2 = 2 or 3, and time on stream = 40 h. The amounts of acid sites are given in Supplementary Tables 4 and 5. STY: space-time yield.

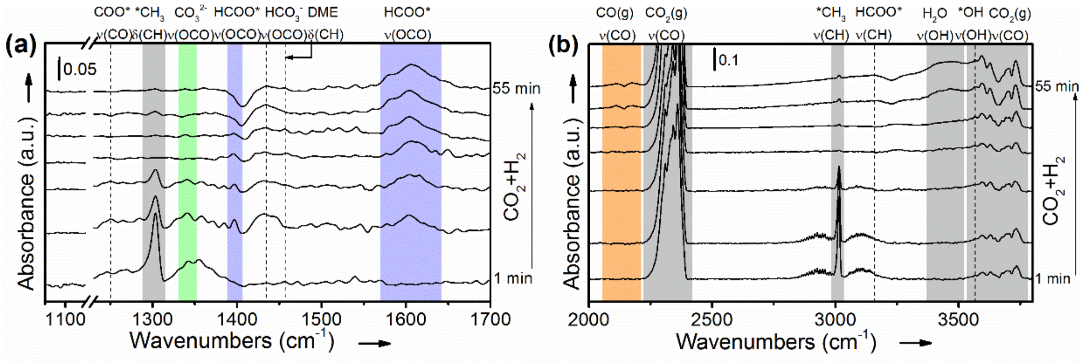

Supplementary Fig. 11. Operando DRIFTS of the CO_2 hydrogenation over GaN-26.6. **(a)** IR spectra in the range of 1050–1700. **(b)** IR spectra in the range of 2000–3600. Conditions: T = 360 ºC, P = 0.1 MPa, H_2/CO_2= 2, and the total flow rate = 20 mL/min.

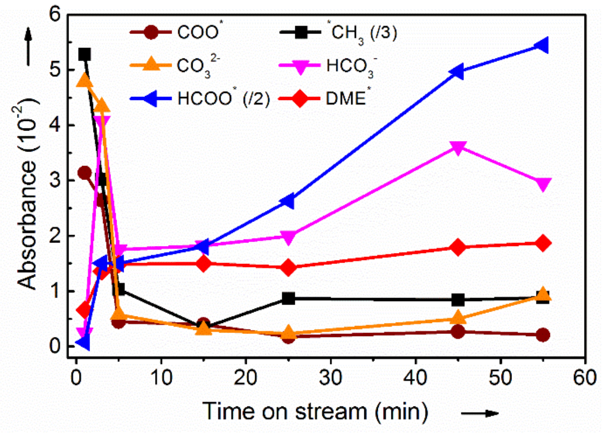

Supplementary Fig. 12. The time-evolution IR absorbance. The species of COO* [ν(C-O) = 1263 cm^{-1}], *CH_3 [ν(C-H) = 3014 cm^{-1}], CO_3^{2-} [ν_s(OCO) = 1340 cm^{-1}], HCO_3^- [ν_s(OCO) = 1430 cm^{-1}], HCOO* [ν_{as}(OCO) = 1606 cm^{-1}] and DME* [δ(C-H) = 1456 cm^{-1}] are analyzed during the CO_2 hydrogenation over the GaN-26.6 catalyst under the conditions of feed of H_2/CO_2 = 2, *P* = 0.1 MPa, and *T* = 360 °C.

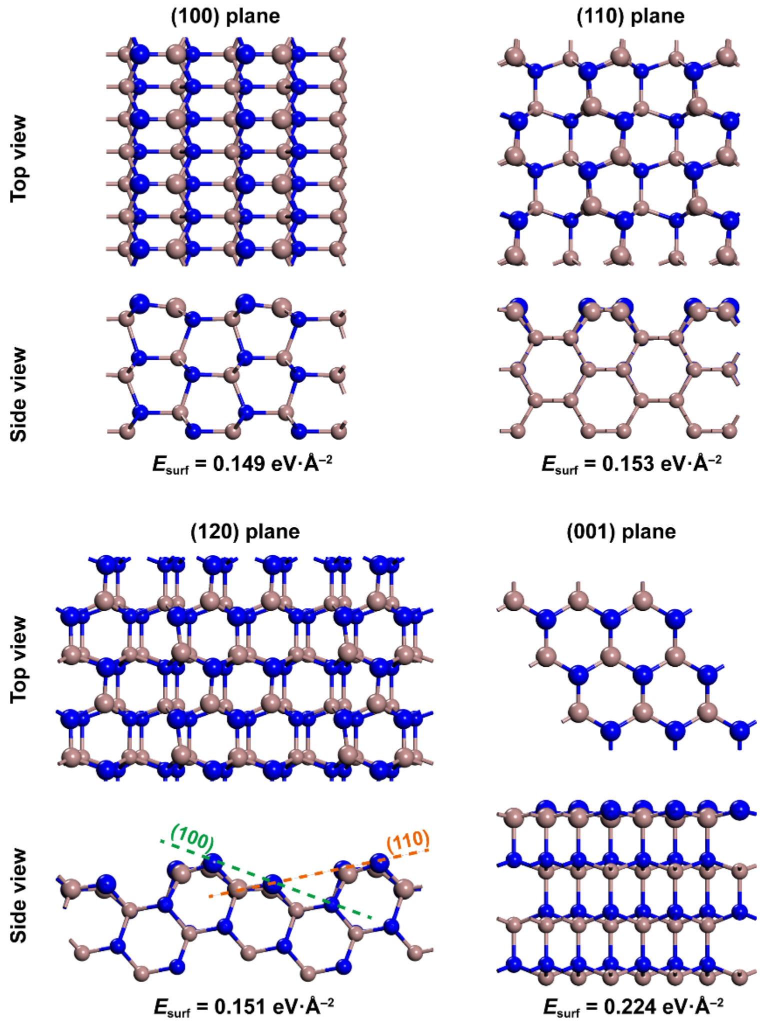

Supplementary Fig. 13. Optimized structure of GaN surfaces. The corresponding surface energies (E_{surf}) are displayed below.

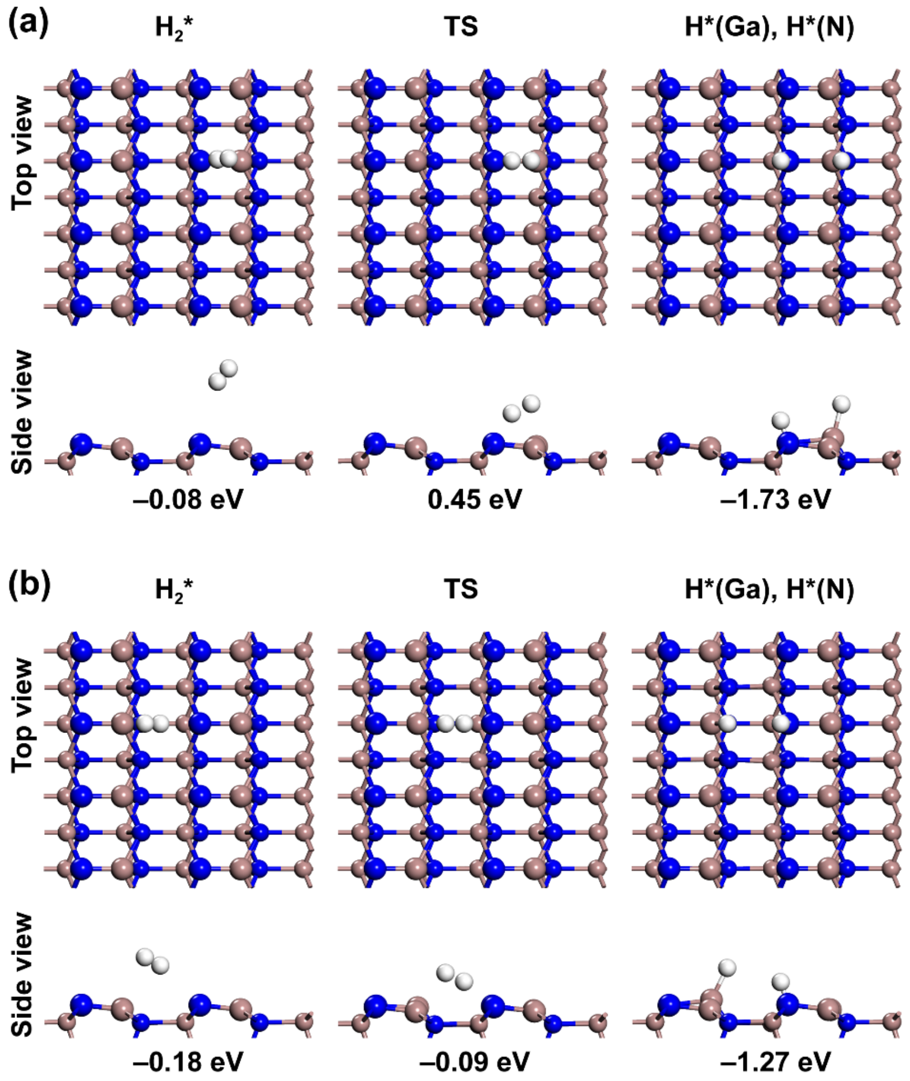

Supplementary Fig. 14. Optimized structures of intermediates and transition states (TS) for the dissociative adsorption of hydrogen on the GaN(100) surface. **(a)** The adsorption of hydrogen at site I. **(b)** The adsorption of hydrogen at site II. The value below each structure is the relative energy of the structure in eV. The zero-energy reference is the sum of H_2 and the corresponding clean surface. The balls in white, blue, and champagne colors represent H, N, and Ga atoms, respectively.

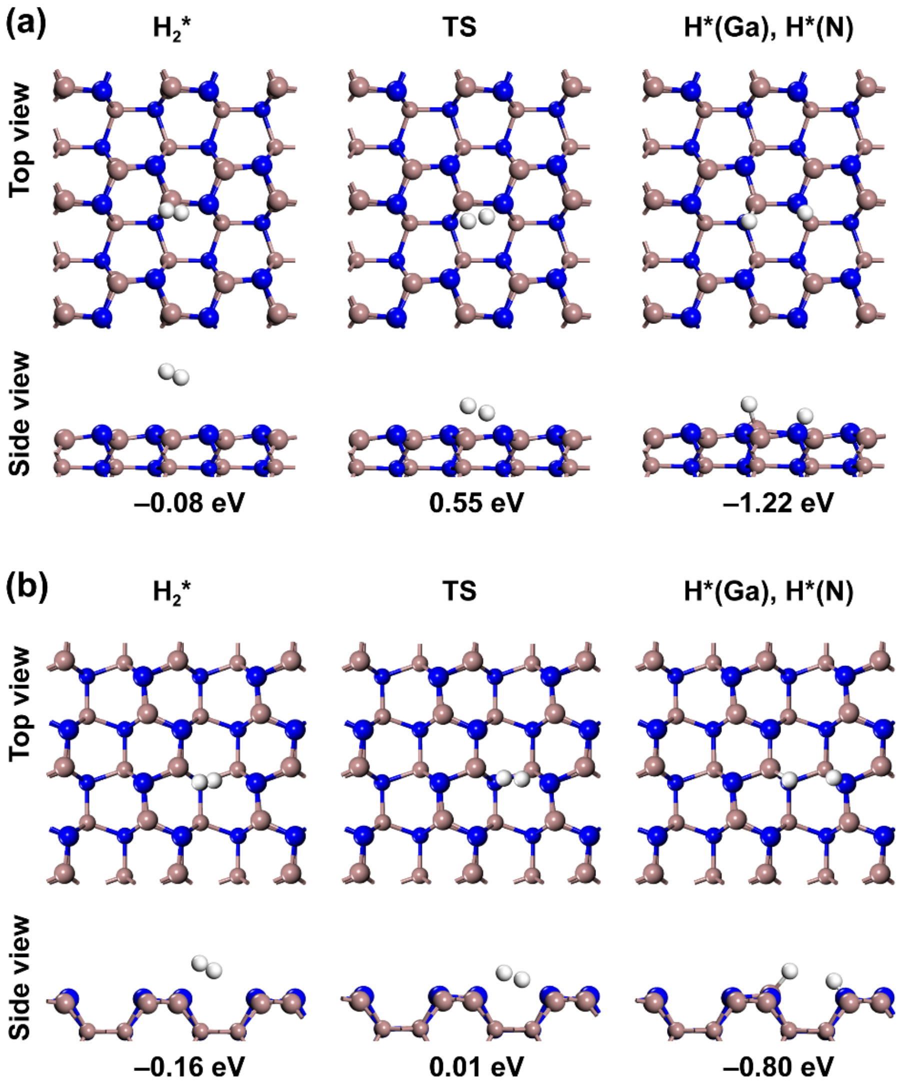

Supplementary Fig. 15. Optimized structures of intermediates and transition states (TS) for the dissociative adsorption of hydrogen on the GaN(110) surface. **(a)** The adsorption of hydrogen at site I. **(b)** The adsorption of hydrogen at site II. The value below each structure is the relative energy of the structure in eV.

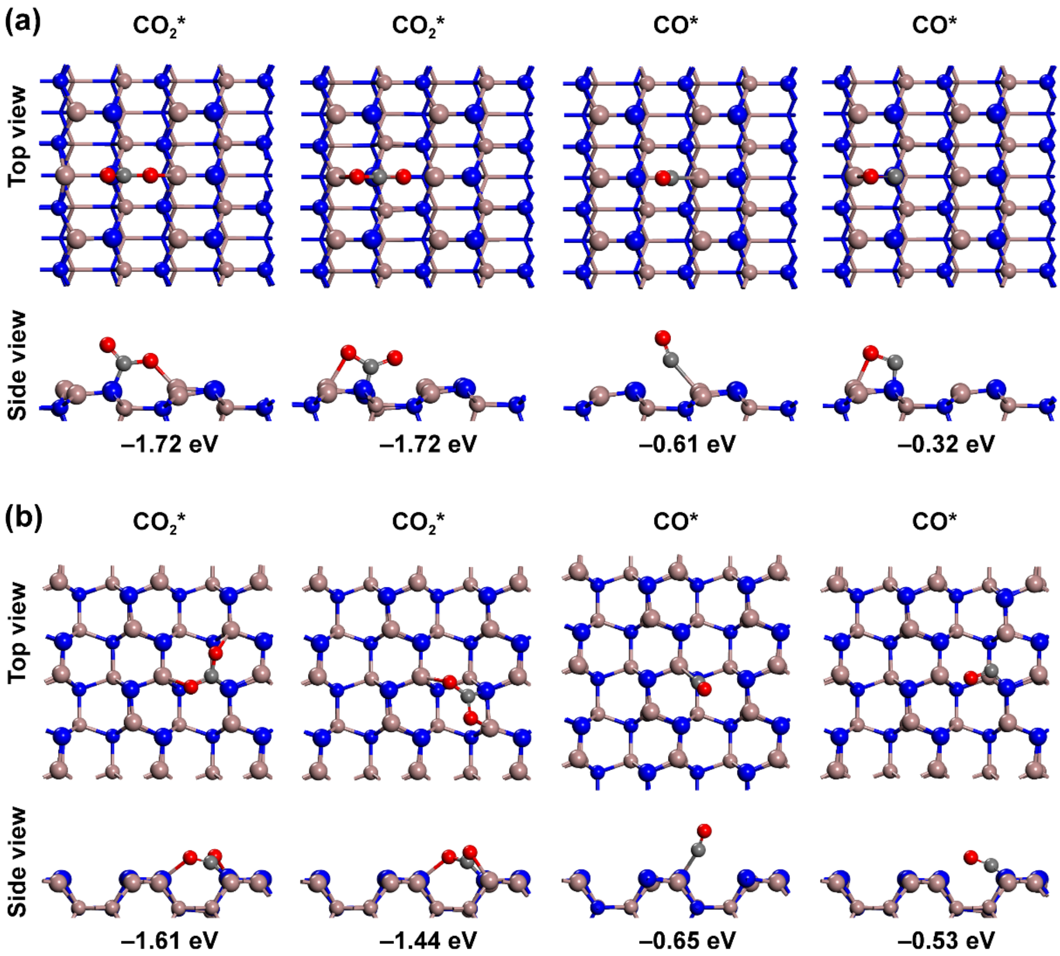

Supplementary Fig. 16. Structure for the optimal adsorption of CO_2 and CO molecules. **(a)** On GaN(100). **(b)** On GaN(110). The value below each structure is the relative energy of the structure in eV. The zero-energy reference is the sum of CO_2 and the corresponding clean surface. The balls in grey, blue, red, and champagne colors represent C, N, O, and Ga atoms, respectively.

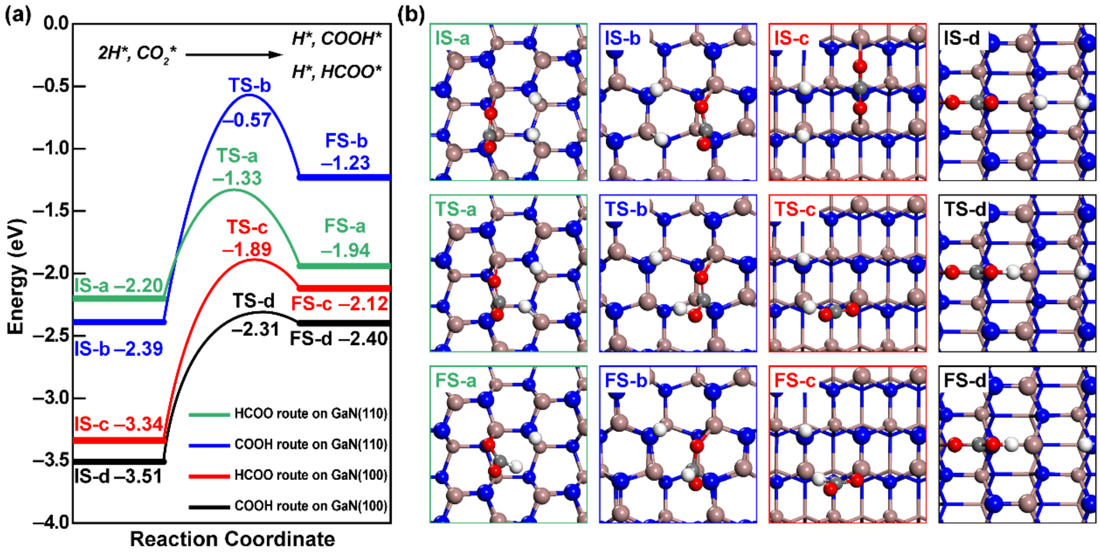

Supplementary Fig. 17. Potential energy profiles and corresponding intermediates and transition states (TS). **(a)** Potential energy profiles of CO_2 hydrogenation to form carboxylate ($COOH^*$) and formate ($HCOO^*$) species on GaN (110) and (100) surfaces. **(b)** Optimized structures of intermediates and TS for the hydrogenation of CO_2 to the intermediates of carboxylate and formate on GaN surfaces. The zero-energy reference is the sum energies of H_2, CO_2, and corresponding clean surfaces.

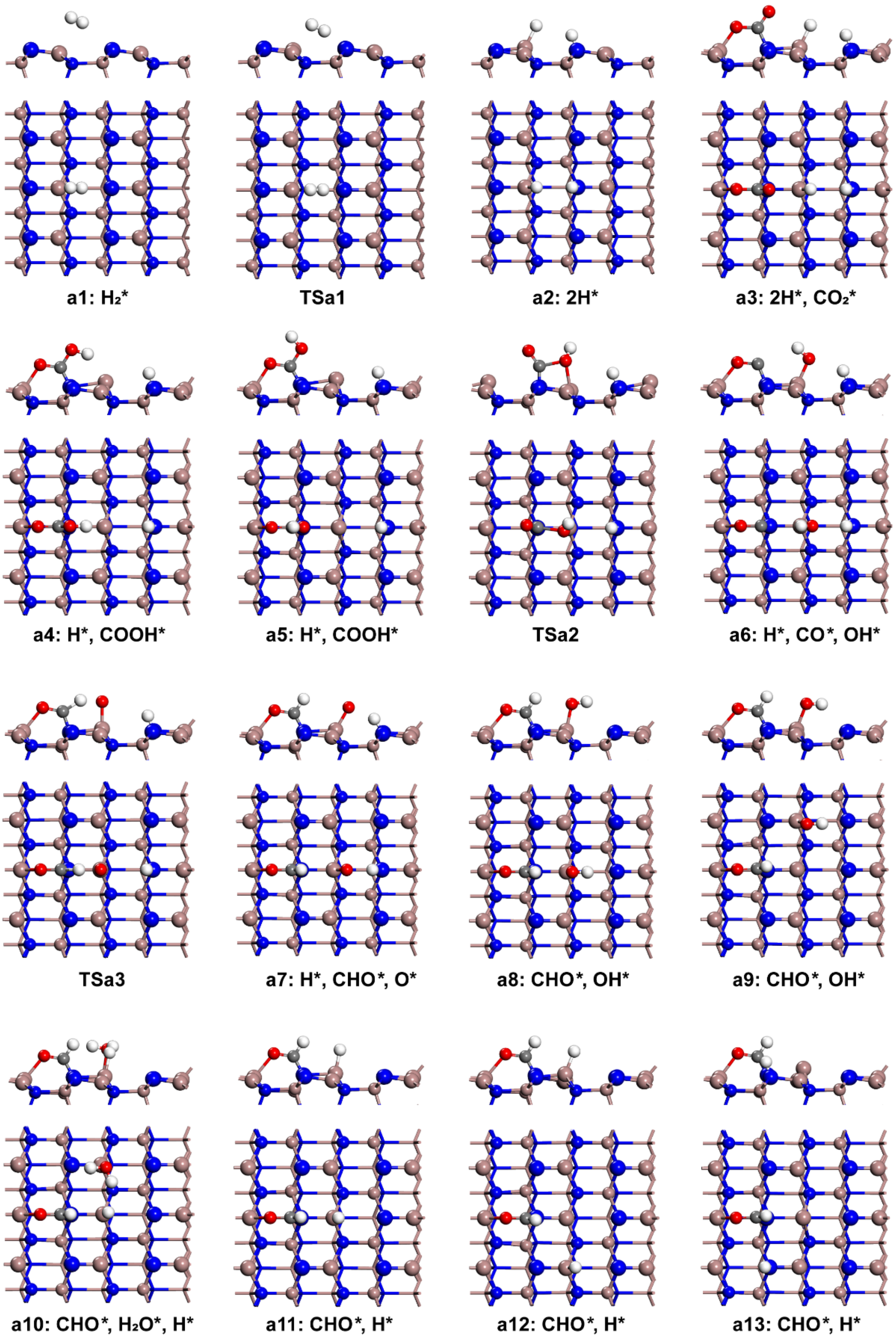

Supplementary Fig. 18. Partial optimized structures for the CO_2 hydrogenation to form methyl on the GaN(100) surface. The structures from a1 to a13 are the intermediates and transition states (TS) in Figure 7a.

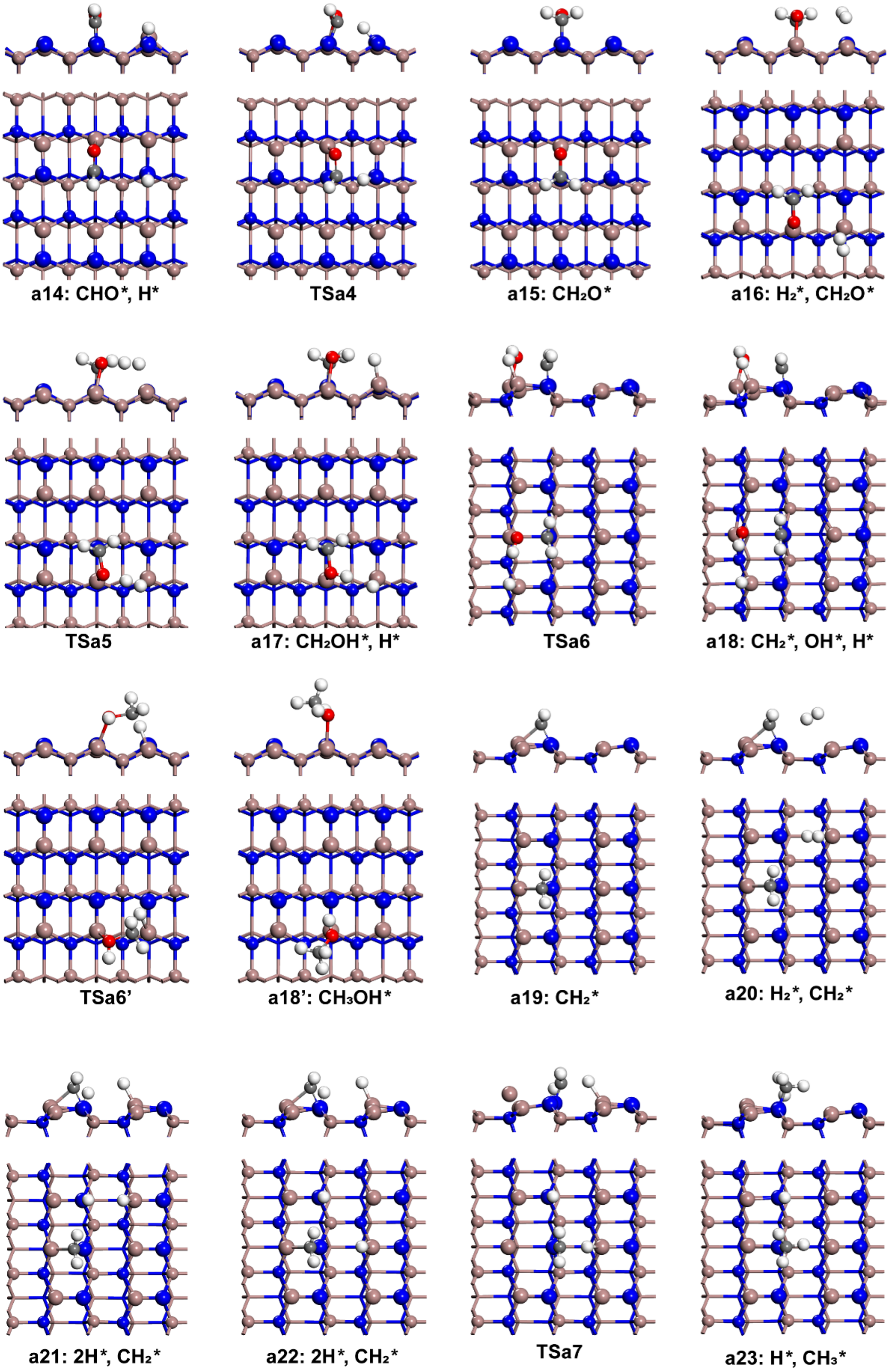

Supplementary Fig. 19. Partial optimized structures for the CO_2 hydrogenation to form methyl on the GaN(100) surface. The structures from a14 to a23 are the intermediates and transition states (TS) in Figure 7a.

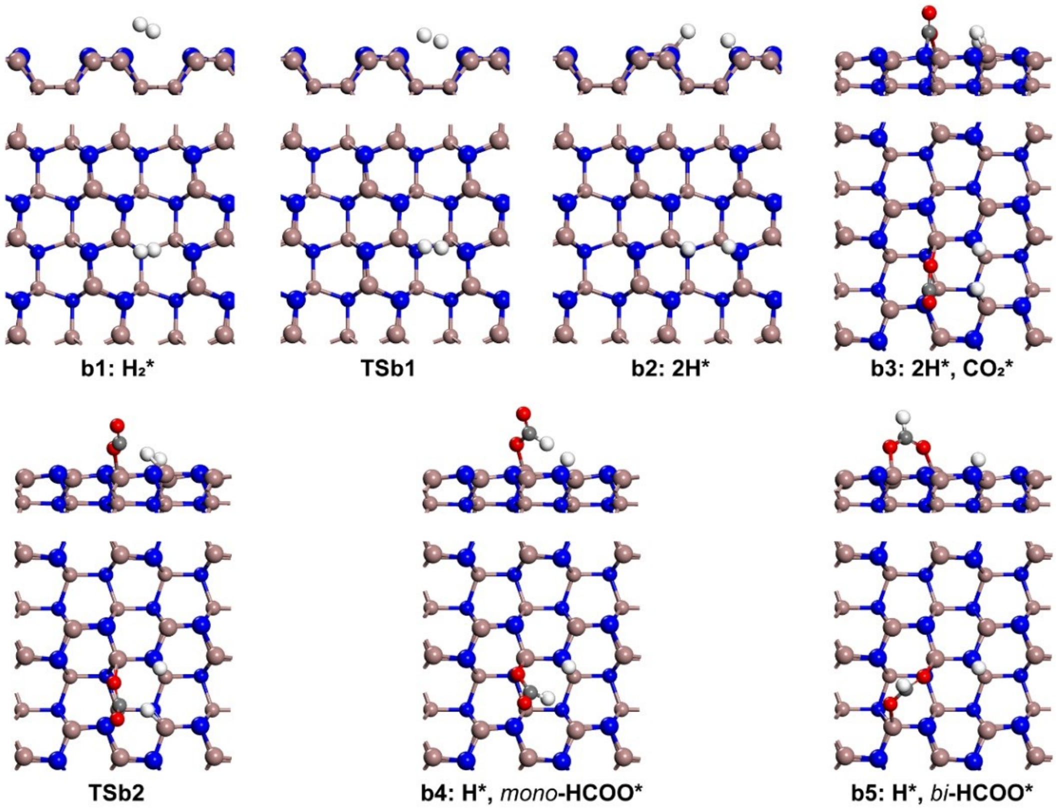

Supplementary Fig. 20. Optimized structures for the CO_2 hydrogenation to generate formate on the GaN(110) surface. The structures from b1 to b5 are the intermediates and transition states (TS) in Figure 7b.

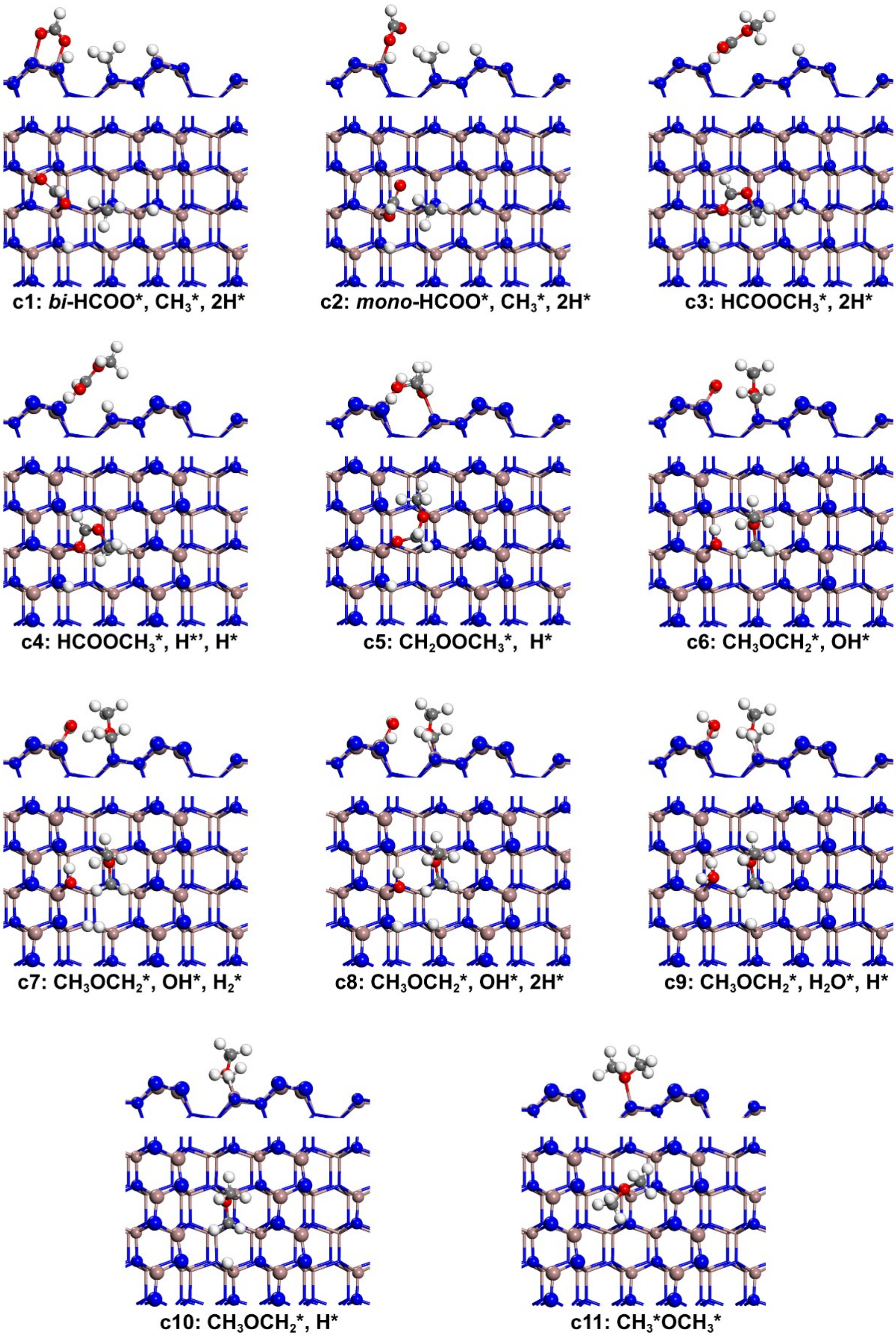

Supplementary Fig. 21. Optimized structures for the formation of DME on the (110)/(100) interface. The structures from c1 to c11 are the intermediates and transition states (TS) in Figure 7c.

Supplementary Table 1. Summary of the binding energies for Ga 2*p*, Ga 3*d* and N 1*s* over different GaN catalysts.

Sample		Fresh GaN-7.4	Fresh GaN-10.5	Fresh GaN-16.7	Fresh GaN-26.6	GaN in reference	Ga_2O_3 in reference
Binding Energy (eV)	Ga $2p_{3/2}$	1117.5	1117.7	1117.9	1117.8	1117.8 [1]	1116.9 [3]
	Ga $2p_{1/2}$	1144.3	1144.5	1144.7	1144.6	1144.6 [1]	1143.7 [3]
	Ga 3*d*	20.0 & 21.1	19.8	20.0	19.8	20.1 [1]<br>19.7 [2]	21.1 [4]
	N 1*s*	397.5	397.4	397.4	397.4	397.4 [2]	-
Sample		Spent GaN-7.4	Spent GaN-10.5	Spent GaN-16.7	Spent GaN-26.6		
Binding Energy (eV)	Ga $2p_{3/2}$	1117.4	1117.8	1117.8	1117.9		
	Ga $2p_{1/2}$	1144.2	1144.6	1144.7	1144.8		
	Ga 3*d*	19.9 & 21.1	20.0	20.0	19.8		
	N 1*s*	397.6	397.5	397.4	397.6		

Supplementary Table 2. The crystal sizes of GaN[a].

Sample	Calcination time (h)			Commercial GaN
	1	2	4	
Crystal size (nm)	7.4	10.5	16.7	26.6

[a] It was obtained based on the Scherrer's formula and the (110) diffraction in Supplementary Fig. 2.

Supplementary Table 3. The acidity of GaN catalysts determined by NH_3-TPD.

Sample	Amount of acid sites ($mmol_{NH3}/g$) [a]		
	Weak	Strong	Total
GaN-7.4	88.9	7.0	95.9
GaN-10.5	28.3	6.9	35.2
GaN-16.7	38.6	6.1	44.7
GaN-26.6	24.1	3.2	27.3
Spent GaN-26.6 [b]	13.5	0.1	13.6

[a] Weak acid sites: NH_3 desorbed in the temperature range of 50–400 °C; Strong acid sites: NH_3 desorbed in the range of higher than 400 °C.

[b] It was obtained after the reaction under the conditions of P = 2.0 MPa, T = 360 ºC, H_2/CO_2 = 2, gas hourly space velocity = 3000 $mL{\cdot}g^{-1}{\cdot}h^{-1}$ and a time on stream of 100 h.

Supplementary Table 4. List for the hydrogenation of CO_2 to DME over the reported Cu-based hybrid catalysts and GaN catalysts

Catalyst	Reaction conditions				Catalytic performance			Reference
	T (ºC)	*P* (MPa)	GHSV ($mL \cdot g^{-1} \cdot h^{-1}$)	H_2/CO_2	CO_2 Conversion (%)	DME Selectivity (%)	STY_{DME} ($mmol \cdot g^{-1} \cdot h^{-1}$)	
$CuZnZrO_x$/Ferrierite	260	5.0	8800	3	26.0	81.3	7.2	[28]
$CuZnZrO_x$/HZSM-5	240	3.0	10000	3	13.4	80.8	4.9	[29]
$CuZnZrO_x$/WO_3-ZrO_2	260	3.0	4333	3	21.5	90.0	5.9	[30]
$CuZnAlO_x$/HZSM-5	240	2.8	1525	3	21.4	86.0	6.2	[31]
$CuZnAlO_x$/HZSM-5	260	3.0	1500	3	19.2	91.6	1.7	[32]
$CuZnAlO_x$/HZSM-5	300	3.0	1800	3	29.5	24.1	0.92	[33]
$CuZrO_x$/Montmorillonite	300	4.0	3600	3	14.1	58.5	0.54	[34]
$CuZnZrO_x$/Y-Zeolite	350	5.0	3000	3	34.1	2.5	0.51	[35]
GaN	360	2.0	3000	3	7.5	75.4	0.56	This work
GaN	360	2.0	3000	2	6.3	79.1	0.85	This work
$CaCO_3$-CaN	360	2.0	3000	2	10.7	47.9	2.9	This work

Supplementary Table 5. Density of acid sites over different GaN catalysts.

Catalyst	Density (μmol/g)		
	Lewis acids	Brønsted acids	Total
GaN-7.4	36.9	44.8	81.7
GaN-10.5	18.7	41.7	60.4
GaN-16.7	26.5	37.0	63.5
GaN-26.6	19.5	6.2	25.7

Supplementary Table 6. The assignment of the IR bands observed in Supplementary Fig. 11.

Species	Formula	Wave numbers (cm^{-1})	References
Carboxylate (COO^{*})	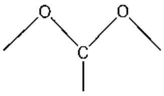	1263 cm^{-1} [ν(C-O)]	[10,11]
Hydroxyl (*OH)	Ga—OH	3600 cm^{-1} [ν(OH)]	[14]
Carbonate (CO_3^{2-})	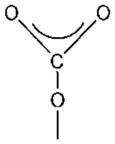	1340 cm^{-1} [ν_s(OCO)]	[12]
Bicarbonate (HCO_3^{-})	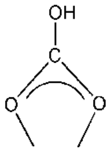	1430 cm^{-1} [ν_s(OCO)]	[17,18,21,22]
Formate ($HCOO^{*}$)	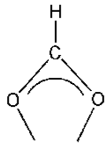	1395 cm^{-1} [ν_s(OCO)]<br>1606 cm^{-1} [ν_{as}(OCO)]<br>3150 cm^{-1} [ν(C-H)]	[17,22]
Absorbed DME<br>($CH_3O^{*}CH_3$)	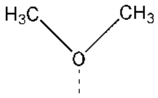	1456 cm^{-1} [δ(C-H)]	[25]
Methyl ($^{*}CH_3$)	—CH_3	1303 cm^{-1} [δ(C-H)]<br>3014 cm^{-1} [ν(C-H)]	[12,13]
Water (H_2O)	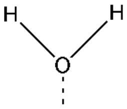	3400 ~ 3550 cm^{-1} [ν(OH)]	[36]
Carbon monoxide<br>(Gaseous CO)	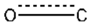	2111 cm^{-1} [ν(C-O)]<br>2171 cm^{-1} [ν(C-O)]	[37]
Carbon dioxide<br>(Gaseous CO_2)	O=C=O	3590 ~ 3730 cm^{-1}	[38]

Supplementary Table 7. Reaction energies (ΔE) and activation energies (E_a) for the elementary steps involved in the hydrogenation of CO_2 on the GaN(110) surface.

Reactions	ΔE (eV)	E_a (eV)
H_2* → H(Ga)* + H*(N) at site I in Supplementary Fig. 15	–1.14	0.63
H_2* → H(Ga)* + H*(N) at site II in Supplementary Fig. 15	–0.64	0.17
H(Ga)* + H*(N) + CO_2* → H*(N) + COOH*	1.16	1.82
H(Ga)* + H*(N) + CO_2* → H*(N) + HCOO*	0.26	0.87

Supplementary Table 8. Reaction energies (ΔE) and activation energies (E_a) for the elementary steps involved in the hydrogenation of CO_2 on the GaN(100) surface.

Reactions	ΔE (eV)	E_a (eV)
H_2* → H(Ga)* + H*(N) at site I in Supplementary Fig. 14	–1.65	0.53
H_2* → H(Ga)* + H*(N) at site II in Supplementary Fig. 14	–1.09	0.09
H(Ga)* + H*(N) + CO_2* → H*(N) + COOH*	1.11	1.20
H(Ga)* + H*(N) + CO_2* → H*(N) + HCOO*	1.22	1.45

Supplementary References

[1] Hao, J. Xu, S. Gao, B. & Pan, L. PL Tunable GaN Nanoparticles Synthesis through Femtosecond Pulsed Laser Ablation in Different Environments. *Nanomaterials* **10,** 439 (2020).

[3] Battistoni, C. Dormann, J. L. Fiorani, D. Paparazzo, E. & Viticoli, S. An XPS and Mössbauer study of the electronic properties of $ZnCr_xGa_{2-x}O_4$ spinel solid solutions. *Solid State Commun.* **39,** 581–585 (1981).

[2] Liu, W-S. Chang, Y-L. Tan, C-Y. Tsai, C-T. & Kuo, H-C. Properties of N-Type GaN Thin Film with Si-Ti Codoping on a Glass Substrate. *Crystals* **10,** 582 (2020).

[4] Wolter, S. D. Luther, B. P. Waltemyer, D. L. Onneby, C.S. Mohney, E. & Molnar, R. J. X-ray photoelectron spectroscopy and X-ray diffraction study of the thermal oxide on gallium nitride. *Appl. Phys. Lett.* **70,** 2156–2158 (1997).

[5] Katsikini, M. Rossner, H. Fieber-Erdmann, M. Holub-Krappe, E. Moustakas, T. D. & Paloura, E.C. Gallium κ-edge EXAFS measurements on cubic and hexagonal GaN. *J. Synchrotron Rad.* **6,** 561–563 (1999).

[6] Chen, H.Y. et al. Preparation of $(Ga_{1-x}Zn_x)(N_{1-x}O_x)$ Photocatalysts from the Reaction of NH_3 with Ga_2O_3/ZnO and $ZnGa_2O_4$: In Situ Time-Resolved XRD and XAFS Studies. *J. Phys. Chem. C.* **113,** 3650–3659 (2009).

[7] Li, L. et al. Thermal Non-Oxidative Aromatization of Light Alkanes Catalyzed by Gallium Nitride. *Angew. Chem. Int. Ed.* **53,** 14106–14109 (2014).

[8] Bao, K. et al. GaN taper rods: Solid-phase synthesis, crystal defects, and optical properties. *J. Solid State Chem.* **181,** 1634–1641 (2008).

[9] Emeis, C. A. Determination of intergrated molar extinction coefficients for infrared absorption bands of pyridine adsorbed on solid acid catalysts. *J. Catal.* **141,** 347–354 (1993).

[10] Wang, F. et al. Active Site Dependent Reaction Mechanism over Ru/CeO_2 Catalyst toward CO_2 Methanation. *J. Am. Chem. Soc.* **138,** 6298–6305 (2016).

[11] Busca, G. & Lorenzelli, V. Infrared spectroscopic identification of species arising from reactive adsorption of carbon oxides on metal oxide surfaces. *I Mat. Chem.* **7,** 89–126 (1982).

[12] Sharma, S. Hu, Z. Zhang, P. McFarland, E. & Metiu, H. CO_2 methanation on Ru-doped ceria. *J. Catal.* **278,** 297–309 (2011).

[13] Zhou, G. et al. Role of surface Ni and Ce species of Ni/CeO_2 catalyst in CO_2 methanation. *Appl. Surf. Sci.* **383,** 248–252 (2016).

[14] Collins, S. Baltanas, M. & Bonivardi, A. An infrared study of the intermediates of methanol synthesis from carbon dioxide over Pd/β-Ga_2O_3. *J. Catal.* **226,** 410–421 (2004).

[15] Jiang, K. Xu, K. Zou, S-Z. & Cai, W-B. B-Doped Pd Catalyst: Boosting Room-Temperature Hydrogen Production from Formic Acid–Formate Solutions. *J. Am. Chem. Soc.* **136,** 4861–4864 (2014).

[16] Miyake, H. Okada, T. Samjeske, G. & Osawa, M. Formic acid electrooxidation on Pd in acidic solutions studied by surface-enhanced infrared absorption spectroscopy. *Phys. Chem. Chem. Phys.* **10,** 3662–3669 (2008).

[17] Le Peltier, F. Chaumette, P. Saussey, J. Bettahar, M. M. & Lavalley, J. C. In situ FT-IR and kinetic study of methanol synthesis from CO_2/H_2 over $ZnAl_2O_4$ and Cu–$ZnAl_2O_4$ catalysts. *J. Mol. Catal. A. Chem.* **132,** 91–100 (1998).

[18] Slostowski, C. Marre, S. Dagault, P. Babot, O. Toupance, T. & Aymonier, C. CeO_2 nanopowders as solid sorbents for efficient CO_2 capture/release processes. *J. CO_2 Util.* **20,** 52–58 (2017).

[19] Shi, L. Yang, G. Tao, K. Yoneyama, Y. Tan, Y. & Tsubaki, N. An Introduction of CO_2 Conversion by Dry Reforming with Methane and New Route of Low-Temperature Methanol Synthesis. *Accounts Chem. Res.* **46,** 1838-1847 (2013).

[20] Lin, L. et al. In Situ Characterization of Cu/CeO_2 Nanocatalysts for CO_2 Hydrogenation: Morphological Effects of Nanostructured Ceria on the Catalytic Activity. *J. Phys. Chem. C* **122,** 12934-12943 (2018).

[21] Weigel, J. Koeppel, R. A. Baiker, A. & Wokaun, A. Surface Species in CO and CO_2 Hydrogenation over Copper/Zirconia: On the Methanol Synthesis Mechanism. *Langmuir.* **12,** 5319–5329 (1996).

[22] Collins, S. E. Chiavassa, D. L. Bonivardi, A. L. & Baltanas, M. A. Hydrogen Spillover in Ga_2O_3–Pd/SiO_2 Catalysts for Methanol Synthesis from CO_2/H_2. *Catal. Lett.* **103,** 83–88 (2005).

[23] Efstathiou, A. M. Chafik, T. Bianchi, D. & Bennett, C.O. A Transient Kinetic Study of the CO/H_2 Reaction on Rh/Al_2O_3 Using FTIR and Mass Spectroscopy. *J. Catal.* **148,** 224–239 (1994).

[24] Yan, Y. Dai, Y. H. He, H. Yu, Y. B. & Yang, Y. H. A novel W-doped Ni-Mg mixed oxide catalyst for CO_2 methanation. *Appl. Catal. B. Environ*. **196,** 108–116 (2016).

[25] Kecskemeti, A. Barthos, R. & Solymosi, F. Aromatization of dimethyl and diethyl ethers on Mo_2C-promoted ZSM-5 catalysts. *J. Catal.* **258,** 111–120 (2008).

[26] Liu, X. Wang, M. Zhou, C. Zhou, W. Cheng, K. Kang, J. Zhang, Q. et al. Selective transformation of carbon dioxide into lower olefins with a bifunctional catalyst composed of $ZnGa_2O_4$ and SAPO-34. *Chem. Commun.* **54,** 113–218 (2018)

[27] Xu, Y. & Ching, W. Electronic, optical, and structural properties of some wurtzite crystals. *Physical Review B.* **48,** 4335–4351 (1993).

[28] Frusteria, F. et al. Direct CO_2-to-DME hydrogenation reaction: New evidences of a superior behaviour of FER-based hybrid systems to obtain high DME yield. *J. CO_2 Util.* **18**, 353–361 (2017).

[29] Frusteri, F. Cordaro, M. Cannilla, C. Bonura, G. Multifunctionality of Cu–ZnO–ZrO_2/H-ZSM5 catalysts for the one-step CO_2-to-DME hydrogenation reaction. *Appl. Catal. B. Environ.* **162**, 57–65 (2015).

[30] Witoon, T. Kidkhunthod, P. Chareonpanich, M. Limtrakul, J. Direct synthesis of dimethyl ether from CO_2 and H_2 over novel bifunctional catalysts containing CuO-ZnO-ZrO_2 catalyst admixed with WO_x/ZrO_2 catalysts. *Chem. Eng. J.* **348**, 713–722 (2018).

[31] Ren, S. et al. Highly active and selective Cu-ZnO based catalyst for methanol and dimethyl ether synthesis via CO_2 hydrogenation. *Fuel.* **239**, 1125–1133 (2019).

[32] Hu, Y. et al. The influence of composition on the functionality of hybrid CuO-ZnO-Al_2O_3/HZSM-5 for the synthesis of DME from CO_2 hydrogenation. *RSC Adv.* **8**, 30387–30395 (2018).

[33] Liu, R. Tian, H. Yang, A. Zha, F. Ding, J. Chang, Y. Preparation of HZSM-5 membrane packed CuO-ZnO-Al_2O_3 nanoparticles for catalysing carbon dioxide hydrogenation to dimethyl ether. *Appl. Surf. Sci.* **345**, 1–9 (2015).

[34] Kornas, A. et al. Direct hydrogenation of CO_2 to dimethyl ether (DME) over hybrid catalysts containing CuO/ZrO_2 as a metallic function and heteropolyacids as an acidic function. *React. Kinet. Mech. Catal.* **130**, 179–194 (2020).

[35] Fujiwara, M. Kieffer, R. Ando, H. Souma, Y. Development of composite catalysts made of Cu-Zn-Cr oxide/zeolite for the hydrogenation of carbon dioxide. *Appl. Catal. A. Gen.* **121**, 113-124 (1995).

[36] Akarmazyan, S. S. Panagiotopoulou, P. Kambolis, A. Papadopouloub, C. D. & Kondarides, I. Methanol dehydration to dimethyl ether over Al_2O_3 catalysts. *Appl. Catal. B. Environ.* **145,** 136–148 (2014).

[37] Bianchi, D. Chafik, T. Khalfallah, M. & Teichner, S. J. Intermediate species on zirconia supported methanol aerogel catalysts: II. Adsorption of carbon monoxide on pure zirconia and on zirconia containing zinc oxide. *Appl. Catal. A. Gen.* **105,** 223–249 (1993).

[38] Chiang, C. L. Lin, K. S. & Lin, Y. G. Preparation and Characterization of Ni_5Ga_3 for Methanol Formation via CO_2 Hydrogenation. *Top. Catal.* **60,** 685–696 (2017).
